# Early Memory and Executive Function as Predictors of Language Development: Evidence for Early Cognitive Foundations in a Taiwanese Cohort

**DOI:** 10.3390/children12111497

**Published:** 2025-11-04

**Authors:** Chiu-Hua Huang, Ishien Li

**Affiliations:** 1 Department of Early Childhood Education, National Pingtung University, Pingtung 90004, Taiwan; hua19@mail.nptu.edu.tw; 2 Department of Early Childhood Education, National Taichung University of Education, Taichung 40306, Taiwan

**Keywords:** memory, executive function, language development, cognitive development, longitudinal study

## Abstract

**Highlights:**

**What are the main findings?**

- Based on longitudinal data from 6652 children in the Kids in Taiwan (KIT) cohort, early memory and executive function (EF) at 12 and 24 months predicted later language outcomes at 24 and 36 months.
- Early memory consistently predicted receptive and expressive language at 24 and 36 months, whereas executive function (EF) significantly predicted expressive language at 24 months and both receptive and expressive language at 36 months.

**What are the implications of the main findings?**

- Behavioral indicators of memory and EF can serve as early, low-cost markers of children’s cognitive foundations for language growth, suitable for use in home and educational settings.
- Integrating these cognitive indicators into developmental screening and early education may improve early identification and intervention for children at risk of language delay.

**Abstract:**

Background: Early cognitive abilities such as memory and executive function (EF) emerge rapidly in infancy and may provide a foundation for later language development. However, large-scale longitudinal evidence linking early cognition to subsequent receptive and expressive outcomes remains limited. Methods: Data were drawn from 6652 children in the Kids in Taiwan (KIT) longitudinal database. Hierarchical regression models tested whether memory and EF at 12 months predicted language comprehension and expression at 24 months, and whether cognition at 24 months predicted outcomes at 36 months, controlling for parental education, involvement, responsiveness, child gender, temperament, and previous language ability. All language variables were standardized to ensure comparability across ages and to minimize potential floor or ceiling effects. Results: Early memory consistently predicted receptive and expressive language at 24 and 36 months, whereas EF predicted expressive language at 24 months and both receptive and expressive language at 36 months. The overall inclusion of cognitive variables significantly increased model fit (all Δ*F*s, *p* < 0.001), indicating that early cognitive functioning contributes uniquely to subsequent language development beyond language stability. Conclusions: Findings from this large community-based Taiwanese cohort highlight the importance of early cognitive abilities in supporting subsequent language growth. Incorporating assessments of memory and EF into early developmental monitoring may help identify children who would benefit from enriched language experiences or targeted educational support. Integrating assessments of memory and EF into early developmental screening and intervention programs may enhance the early identification of children at risk for delayed language development and guide the design of play-based activities that strengthen cognitive foundations for language.

## 1. Introduction

Early language development during infancy and toddlerhood lays the foundation for later literacy and academic achievement [1,2]. Family and caregiving environments play a critical role in shaping these outcomes, particularly through parental responsiveness, involvement, and caregiver–child interactions [3,4]. Sustained parental responsiveness across early childhood predicts stronger language skills in middle childhood [3], and positive, high-quality caregiver engagement promotes vocabulary and comprehension even in high-risk contexts [5].

Temperament, defined as biologically based individual differences in reactivity and self-regulation [6], also contributes to language variation. Higher effortful control is associated with greater expressive vocabulary and self-regulatory competence [6], while surgency—reflecting sociability and positive affectivity—facilitates conversational engagement and vocabulary growth [7,8]. In contrast, heightened negative affectivity may constrain language learning, particularly for children who experience fewer responsive interactions [9,10]. Because temperament may influence both cognitive engagement and linguistic exposure, the present study includes these dimensions as covariates.

Beyond temperament, emerging evidence conceptually links early cognitive capacities—such as memory and executive function (EF)—to the development of neural systems that support learning and communication. Neuroimaging studies show that the hippocampus is functionally active within the first year of life, supporting statistical learning and sequence binding [11], and that rapid prefrontal growth enables emerging EF processes such as attention regulation and inhibition [12]. Together, these capacities help infants encode, retain, and flexibly use linguistic information, providing a cognitive foundation for later language development [13].

Structural connectivity further contributes to these developmental processes. Infants with more organized white-matter pathways—including the arcuate fasciculus, corpus callosum, and inferior fronto-occipital fasciculus—show stronger receptive and expressive skills in later years [14,15,16]. Early conversational experience also predicts greater myelination in these tracts [17], underscoring that environmental input interacts with neurodevelopment to shape language outcomes. Collectively, these findings suggest that early memory and EF reflect functional capacities theoretically associated with hippocampal- and prefrontal-supported learning, even though behavioral measures alone do not directly index neural maturation.

Most previous evidence on these links has come from small-sample neuroimaging studies or cross-sectional designs, limiting generalizability. Large, population-based longitudinal data are needed to determine whether early cognitive indicators predict later language outcomes across typical variation in families and children.

Addressing this gap, the present study draws on the nationally representative Kids in Taiwan (KIT) cohort to examine whether early cognitive functioning predicts later language development. Specifically, we test whether memory and EF at 12 months predict language comprehension and expression at 24 months, and whether these cognitive abilities at 24 months predict language outcomes at 36 months, while controlling for parental background and child temperament. By focusing on theoretically grounded behavioral indicators of early cognitive functioning, this study aims to clarify how foundational memory and EF processes contribute to the developmental pathways of early language.

## 2. Literature Review

### 2.1. Early Language Development and Environmental Predictors

Language development in the first three years of life lays the foundation for later literacy, social competence, and academic achievement [1,2]. A substantial body of research has documented the importance of environmental influences, including both family and childcare settings, in shaping children’s language outcomes. Parental responsiveness, involvement, and education levels consistently predict vocabulary and comprehension growth, while caregiver–child interactions in childcare contexts provide additional scaffolding for language learning [3,4,5]. For example, longitudinal studies demonstrate that sustained parental responsiveness is associated with stronger language skills [3], and high-quality verbal exchanges with caregivers can buffer children in high-risk contexts from poorer language outcomes [4,5]. Together, these findings underscore family background and parental engagement as foundational contextual factors in children’s language development.

### 2.2. Child Temperament and Language Development

Building on the role of environmental influences, children’s own characteristics also shape early language trajectories. Temperament, defined as biologically based individual differences in reactivity and self-regulation [6], has also been linked to language development. Children who score higher in effortful control are more likely to sustain attention and regulate impulses, thereby facilitating participation in language-rich interactions and predicting stronger language outcomes [18,19]. Surgency, reflecting sociability and positive affect, promotes engagement in conversational exchanges and more advanced expressive skills [7,8].

Conversely, high negative affectivity may constrain language growth, particularly when caregiving is limited [9,10]. Because temperament may confound associations between cognition and language, the present study includes these dimensions as covariates.

### 2.3. Cognitive Foundations: Memory and Executive Function

Early language development is grounded not only in contextual and temperamental factors but also in children’s emerging cognitive capacities. Two processes—memory and executive function (EF)—form the core cognitive foundation that supports language growth.

Early memory development is closely tied to the emergence of vocabulary and comprehension skills. Longitudinal research has further shown that verbal comprehension and processing speed predict improvements in fact memory among 5- and 6-year-olds [20]. Neurocognitive studies provide converging evidence. The hippocampus is functionally active within the first year of life, supporting recognition memory and statistical learning—the ability to detect recurring patterns in speech and environmental input [11]. These processes help children extract and store linguistic regularities, thereby scaffolding vocabulary acquisition [1].

Everyday behavior also illustrates the role of memory in language. Young children recall storybooks, name caregivers, and create short imaginative narratives. Research has consistently shown that stronger memory skills predict richer narrative abilities—for example, children with better recall produce more coherent stories and show better long-term language outcomes [21].

While memory supports storage and retrieval, EF provides the regulatory skills necessary to use language effectively. During infancy and toddlerhood, the prefrontal cortex undergoes rapid development, enabling EF processes such as attentional regulation, inhibitory control, and working memory [12,22]. To avoid neuroessentialism, references to prefrontal maturation are framed as supporting functional development of self-regulatory abilities. Sensitive caregiving also fosters EF by attention regulation and self-control [23].

In everyday life, EF is reflected in children’s ability to follow requests, suppress prohibited behaviors, and sustain attention skills that facilitate conversation and comprehension. Empirical studies support this role; working memory and inhibition abilities are associated with vocabulary knowledge and story comprehension [24,25,26].

In sum, memory enables children to encode and retrieve linguistic information, while EF allows them to regulate attention and flexibly use language. Both capacities emerge through developmental and environmental processes.

### 2.4. Neural Mechanisms of Early Speech and Language

Behavioral studies highlight the importance of memory and EF for language development, while neuroimaging findings provide complementary insights into how the infant brain processes speech.

Young infants engage cortical networks to discriminate speech sounds and show stronger activation to the mother’s voice [27,28]. Such findings indicate early specialization for speech perception. Furthermore, electrophysiological studies reveal that auditory speech sounds activate corresponding motor regions even before babbling, suggesting early coupling between perception and production [29]. Together, these findings suggest that neural specialization for speech develops in parallel with, and may support, emerging behavioral capacities for perception and communication.

### 2.5. Conceptual Framework and Hypotheses

The present study is guided by a conceptual framework in which young children’s cognitive abilities are conceptualized as behavioral indicators of emerging neurocognitive process, rather than direct neural measures. Memory is interpreted as reflecting hippocampal-supported processes of encoding and retrieval, and EF as reflecting attention and regulation, skills associated with prefrontal development. These constructs are theoretically—but not physiologically—linked to the emergence of early language networks.

Based on this framework, we formulated two hypotheses:

**H1:**  *Memory and executive function (EF) at 12 months will independently predict children’s language comprehension and expression at 24 months.*

**H2:**  *Memory and executive function (EF) at 24 months will independently predict children’s language comprehension and expression at 36 months.*

These hypotheses are interpreted within a developmental context informed by neuroscience, while recognizing that the present measures reflect observable behavioral functioning.

### 2.6. The Present Study

In summary, prior research highlights three major domains that jointly influence children’s early language development: environmental inputs, child temperament, and cognitive foundations such as memory and executive function.

While previous studies have provided valuable insights, most have relied on small samples or cross-sectional designs, limiting generalizability. To advance this field, large-scale longitudinal data are needed to clarify whether early behavioral indicators of cognition predict subsequent language outcomes across typical developmental variation.

The present study addresses this gap by drawing on data from the nationally representative Kids in Taiwan (KIT) cohort. Specifically, we investigate whether memory and executive function at 12 months predict language comprehension and expression at 24 months, and whether these cognitive skills at 24 months predict language outcomes at 36 months, controlling for parental variables and child temperament.

**Figure 1:**
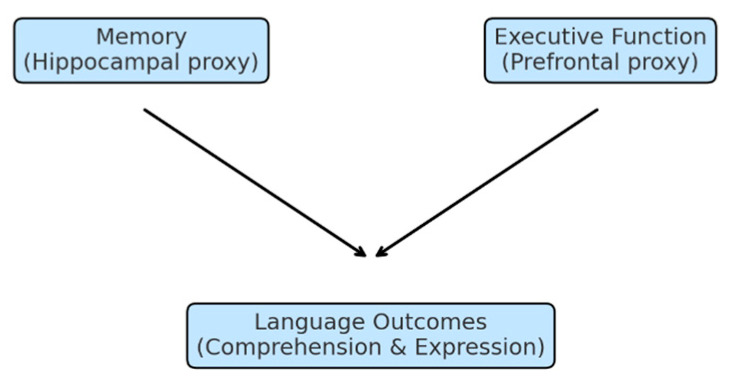
Conceptual framework illustrating how early memory (hippocampal-related behavioral indicator) and executive function (prefrontal-related behavioral indicator) predict later language comprehension and expression.

By situating early memory and EF as behavioral indicators theoretically linked to early neurocognitive processes, this study integrates population-level behavioral evidence with developmental theory to clarify the pathways through which early cognition supports language growth (Figure 1).

## 3. Methods

### 3.1. Participants and Dataset

This study used data from the Kids in Taiwan: National Longitudinal Study of Child Development and Care (KIT) project, a government-supported cohort designed to examine children’s development, family environments, and childcare contexts in Taiwan [30]. KIT employed a two-stage stratified random sampling method, first selecting districts and townships proportional to population size and then randomly selecting children from household registration records, thereby ensuring that the sample represents Taiwan’s demographic diversity. Approximately 6800 children were recruited and have been followed longitudinally through parental interviews and developmental assessments.

The present analysis focused on the KIT M3 cohort assessed at 12, 24, and 36 months. Valid language data were available for 6874 children at 12 months, 6775 at 24 months, and 6652 at 36 months. Parental questionnaires provided information on family background and parenting variables, while primary caregivers completed child developmental items.

The KIT project was approved by the National Chung-Hua University of Education Research Ethics Committee (NCUE-REC-110-031). Written informed consent was obtained from parents or legal guardians before each wave of data collection. Participation was voluntary, and confidentiality and withdrawal rights were fully guaranteed, ensuring adherence to ethical standards.

The KIT data are publicly archived at the Survey Research Data Archive (SRDA), Academia Sinica, Taiwan. The KIT-M3 cohort, recruited at 3 months of age, was analyzed using waves 3 to 5 (12, 24, and 36 months). The dataset (accession D00180) [30] is available from the SRDA (https://doi.org/10.6141/TW-SRDA-D00180-2) under institutional access regulations (accessed on 22 March 2024).

#### Sample Attrition and Missing Data Handling

Attrition across the KIT waves was modest. Attrition across the KIT waves was modest. Attrition across waves was modest: from 6874 cases at 12 months to 6775 at 24 months (≈1.4%) and 6652 at 36 months (≈1.8%). Overall attrition between 12 and 36 months was ≈3.2%. Because missingness was small and largely limited to parental variables, listwise deletion was applied. Multiple imputation was considered but deemed unnecessary.

### 3.2. Measures

All measures were derived from the KIT survey and developmental assessments. Appendix A summarizes constructs, sample items, score ranges, and reliability indices.

- Language outcomes. At 12, 24, and 36 months, children’s receptive and expressive language abilities were assessed with caregiver-reported developmental items. Separate indicators were created for comprehension and expression at each age.
- Memory. At 12 and 24 months, memory was assessed through caregiver reports of recognition, recall, and representational flexibility items (e.g., recalling familiar stories, naming familiar people). Although brief, these items capture early memory-related behaviors shown to predict subsequent cognitive and language outcomes.
- Executive function (EF). EF was measured at 12 and 24 months using items reflecting inhibitory control, attention shifting, and working memory (e.g., stopping a prohibited action when asked, sustaining focus on a task). These measures align with validated EF frameworks in early development [22].
- Family background. Parental (paternal and maternal) education levels were reported in years of schooling. Parental (paternal and maternal) involvement and Parental responsiveness were measured with validated questionnaire items. Parental education level was coded on a 6-point ordinal scale (1 = Elementary school or below, 6 = Master’s degree or above). For the purposes of statistical analyses, it was treated as a continuous variable, and descriptive statistics are reported as means and standard deviations.
- Temperament. Surgency, effortful control, and negative affectivity were measured with caregiver-reported items adapted from Rothbart’s Infant Behavior Questionnaire. Internal consistency for multi-item measures ranged from acceptable to good (Cronbach’s α = 0.71–0.83). These dimensions were included as covariates given their known associations with both cognition and language [6].

### 3.3. Analytic Strategy

Analyses proceeded in two stages. First, descriptive statistics and correlations were computed. Second, hierarchical regression models examined whether memory and EF at 12 months predicted language comprehension and expression at 24 months, and whether 24-month cognition predicted 36-month outcomes. All models controlled for parental education, involvement, and responsiveness, child temperament (effortful control, surgency, negative affectivity), prior language ability, and child gender (male = 1, female = 0). All language scores were standardized (z scores) to ensure comparability across ages and reduce floor/ceiling effects. Model assumptions were verified (all VIFs < 2; residuals approximately normal and homoscedastic). Standardized βs, 95% CIs, and Cohen’s f^2^ effect sizes were reported. Sensitivity analyses excluding cases with missing paternal data yielded consistent results.

## 4. Results

### 4.1. Descriptive Statistics and Correlations of Study Variables

Table 1 displays the means and standard deviations of memory, executive function, language comprehension, and language expression at 36 months, indicating that children’s average language scores are higher than their cognitive scores.

Table 2 presents that the gender distribution of participants at 36 months is relatively balanced, with a nearly 1:1 ratio of male to female; the distributions of paternal and maternal education levels are also similar.

Table 3 shows correlations among memory, executive function, language comprehension, and language expression at 12, 24, and 36 months in this study. Correlations between memory and language were particularly high at 36 months (r ≈ 0.76), reflecting the close linkage between cognitive and linguistic skills at this stage.

### 4.2. Predicting 24-Month Outcomes from 12-Month Cognition

#### 4.2.1. Hierarchical Regression Predicting 24 Month Language Comprehension from 12-Month Cognition

The results of the hierarchical regression are displayed in Table 4. The analyses examine predictors of children’s language comprehension at 24 months. In Step 1, the set of five control variables accounted for a significant proportion of variance, *R*^2^ = 0.053, *F*(5, 5547) = 61.94, *p* < 0.001, and all five variables were significant predictors. In Step 2, the addition of child characteristics (gender, surgency, effortful control, and negative affectivity) and language comprehension at 12 months explained an additional 10.1% of variance, Δ*R*^2^ = 0.101, *F*(5, 5542) = 131.79, *p* < 0.001, increasing the total explained variance to 15.4%, *F*(10, 5542) = 100.51, *p* < 0.001. At this stage, paternal education, maternal education, paternal involvement, and parental responsiveness remained significant predictors, whereas maternal involvement was no longer significant. Among the newly added variables, child gender, surgency, effortful control, and language comprehension at 12 months were significant predictors, whereas negative affectivity was not.

In Step 3, memory and executive function were entered, explaining an additional 2.4% of variance, Δ*R*^2^ = 0.024, *F*(2, 5540) = 79.98, *p* < 0.001, and increasing the total explained variance to 17.7%, *F*(12, 5540) = 99.48, *p* < 0.001. In the final model, the results showed that maternal involvement and negative affectivity remained non-significant. In the final model, language comprehension at 12 months (*β* = 0.172, *p* < 0.001), memory (*β* = 0.187, *p* < 0.001), and executive function (*β* = 0.024, *p* = 0.129) were retained in the final model, with memory emerging as a significant predictor whereas EF was non-significant. The standardized coefficient for language comprehension at 12 months decreased from *β* = 0.254 to *β* = 0.172 after the inclusion of cognitive predictors. Memory was a significant predictor of 24-month language comprehension, whereas executive function did not reach statistical significance.

Taken together, these findings reveal three key patterns. First, language comprehension demonstrates cross-age stability. Even after controlling for family background and child characteristics, language comprehension at 12 months remained a significant predictor of language comprehension at 24 months. Second, when cognitive predictors (memory and executive function) were included, the predictive strength of 12-month language comprehension decreased, showing that part of this stability can be explained by early cognitive mechanisms. In particular, early memory ability appears to provide additional cognitive scaffolding for later language comprehension development. Finally, executive function at 12 months is likely still in a developmental stage; thus, its influence on later language comprehension was not evident at this age.

#### 4.2.2. Hierarchical Regression Predicting 24-Month Language Expression from 12-Month Cognition

Table 5 presents results from hierarchical regressions examining predictors of children’s language expression at 24 months. Step 1, the set of five control variables accounted for a significant proportion of variance, *R*^2^ = 0.082, *F*(5, 5544) = 99.12, *p* < 0.001, and all variables except maternal involvement were significant predictors.

In Step 2, child characteristics and prior language (i.e., language expression at 12 months) were added, explaining an additional 8.2% of variance, Δ*R*^2^ = 0.082, *F*(5, 5539) = 109.13, *p* < 0.001, and increasing the total explained variance to 16.4%, *F*(10, 5539) = 108.96, *p* < 0.001. Among the family background variables entered in Step 1, paternal education, maternal education, paternal involvement, and parental responsiveness remained significant, whereas maternal involvement continued to be nonsignificant. Among the newly added child-level variables, gender, surgency, effortful control, and prior language expression were significant predictors, while negative affectivity was not.

In Step 3, memory and executive function were added to the model, explaining an additional 3.7% of the variance, Δ*R*^2^ = 0.037, *F*(2, 5537) = 127.85, *p* < 0.001, and increasing the total explained variance to 20.1%, *F*(12, 5537) = 116.27, *p* < 0.001. Maternal involvement and negative affectivity remained nonsignificant. In the final model, memory showed a strong positive association with language expression at 24 months (*β* = 0.203, *p* < 0.001), whereas executive function also contributed significantly but with a smaller effect (*β* = 0.048, *p* = 0.002). The standardized coefficient for prior language expression decreased from *β* = 0.097 to *β* = 0.066 after cognitive predictors were added, indicating that part of the continuity in language development is explained by early cognitive abilities.

Overall, the results indicate the stability of language expression ability across different ages. When cognitive variables (memory and executive function) were included in the model, the predictive power of language expression at 12 months decreased, indicating that part of this stability can be explained by early cognitive mechanisms. Notably, although both early memory and executive function significantly predicted later language expression, memory showed a stronger positive predictive effect compared to executive function.

### 4.3. Predicting 36-Month Outcomes from 24-Month Cognition

#### 4.3.1. Hierarchical Regression Predicting 36-Month Language Comprehension from 24-Month Cognition

Table 6 presents the results of the hierarchical regression analyses, which were conducted to examine predictors of children’s language comprehension at 36 months. Step 1, the set of five control variables accounted for a significant proportion of the variance, *R*^2^ = 0.026, *F*(5, 6254) = 33.598, *p* < 0.001, and only paternal involvement and parental responsiveness were significant predictors. In Step 2, adding child characteristics and language comprehension at 24 months explained an additional 12.6% of the variance, Δ*R*^2^ = 0.126, *F*(5, 6249) = 185.29, *p* < 0.001, increasing the total explained variance to 15.2%, *F*(10, 6249) = 111.92, *p* < 0.001. Among the family background variables, only paternal involvement remained significant. In addition, child gender (*β* = 0.040, *p* = 0.001), surgency (*β* = 0.098, *p* < 0.001), effortful control (*β* = 0.131, *p* < 0.001), and language comprehension at 24 months (*β* = 0.247, *p* < 0.001) significantly predicted language comprehension at 36 months. In Step 3, the addition of memory and executive function explained an additional 2.9% of the variance, Δ*R*^2^ = 0.029, *F*(2, 6247) = 110.71, *p* < 0.001, increasing the total explained variance to 18.1%, *F*(12, 6247) = 114.99, *p* < 0.001. Among all predictors in the final model, memory (*β* = 0.159, *p* < 0.001) and executive function (*β* = 0.119, *p* < 0.001) significantly predicted language comprehension at 36 months. Notably, the predictive strength of language comprehension at 24 months decreased from *β* = 0.247 to *β* = 0.162 after the inclusion of cognitive variables, although it remained significant (*p* < 0.001).

Taken together, these findings suggest that early language comprehension also exhibit longitudinal continuity, yet this continuity is further supported by underlying cognitive mechanisms. When memory and executive function were added to the model, the predictive strength of early language comprehension declined, and memory, in particular, played a key role in shaping later language comprehension performance.

#### 4.3.2. Hierarchical Regression Predicting 36-Month Language Expression from 24-Month Cognition

Results from 24 to 36 months largely mirrored the earlier pattern, with both memory and EF showing significant effects on later language outcomes. Table 7 presents the hierarchical regression analyses predicting expressive language at 36 months. Step 1, the set of five control variables accounted for a significant proportion of variance, *R*^2^ = 0.096, *F*(5, 6250) = 133.35, *p* < 0.001, and all five variables were significant predictors. In Step 2, adding child characteristics and language expression at 24 months explained an additional 22.3% of the variance, Δ*R*^2^ = 0.223, *F*(5, 6245) = 409.50, *p* < 0.001, increasing the total explained variance to 32%, *F*(10, 6245) = 293.22, *p* < 0.001. Among these predictors, only maternal involvement and negative affectivity were non-significant, whereas all other variables were significant predictors. Specifically, child gender (*β* = 0.030, *p* = 0.005), surgency (*β* = 0.061, *p* < 0.001), effortful control (*β* = 0.105, *p* < 0.001), and language expression at 24 months (*β* = 0.424, *p* < 0.001) significantly predicted language expression at 36 months.

In Step 3, the addition of memory and executive function explained an additional 1.8% of the variance, Δ*R*^2^ = 0.018, *F*(2, 6243) = 85.24, *p* < 0.001, increasing the total explained variance to 33.8%, *F*(12, 6243) = 265.15, *p* < 0.001. Among all predictors in the final model, memory (*β* = 0.137, *p* < 0.001) and executive function (*β* = 0.096, *p* < 0.001) significantly predicted language expression at 36 months. Notably, the predictive strength of language expression at 24 months decreased from *β* = 0.424 to *β* = 0.330 after the inclusion of cognitive variables, although it remained significant (*p* < 0.001).

Taken together, these findings indicate that early expressive language also exhibit longitudinal stability. Although early language expression remained a significant predictor of later outcomes, its predictive strength decreased after memory and executive function were added to the model, further suggesting that cognitive abilities provide additional explanatory power for subsequent language development.

## 5. Discussion

### 5.1. Main Findings

This study examined how early cognitive abilities—specifically memory and executive function (EF)—contribute to later language comprehension and expression after accounting for family and child characteristics. Across the longitudinal models, both memory and EF predicted later language outcomes, with memory showing a somewhat stronger effect. EF was not associated with 24-month language comprehension but showed significant effects on 24-month expression and both comprehension and expression at 36 months. The contribution of earlier language ability declined after memory and EF were added to the models, suggesting that early cognitive mechanisms partly account for the observed continuity in language development. Taken together, these findings indicate that early cognitive abilities form an enduring foundation for language growth, even as their specific contributions gradually diminish with increasing environmental and experiential influences.

Building on these findings, early language and domain-general cognitive abilities appear closely intertwined during development. The continuity between earlier and later language may partly reflect concurrent memory and executive control capacities. These processes help maintain stability in language development and serve as foundational mechanisms through which early linguistic skills support later growth. This interpretation accords with developmental frameworks proposing that stability in language over time reflects the ongoing maturation of general cognitive systems such as working memory and executive control [31,32]. Recent evidence supports this view: in a systematic review, Bal et al. [33] reported moderate correlations between language development and EF in early childhood, suggesting reciprocal and mutually reinforcing relations between linguistic and cognitive growth.

### 5.2. Developmental Interpretation

Predictive associations between early cognition and later language emerged clearly during the transition from infancy to toddlerhood (12–24 months) and remained robust from 24 to 36 months. During the first period, memory showed a particularly strong predictive effect on expressive language, highlighting the role of representational memory in supporting children’s ability to retrieve and use linguistic forms. From 24 to 36 months, both memory and executive function (EF) significantly predicted later language outcomes, indicating that language development becomes increasingly linked to the coordination of multiple cognitive processes. These findings suggest that early memory may provide the initial foundation for language expression, while EF later contributes additional support as children engage in more complex communicative and self-regulatory tasks.

This developmental pattern aligns with accounts emphasizing the joint maturation of representational and control systems during early childhood. Memory behaviors, such as recognition and recall, facilitate the retention and retrieval of linguistic information, whereas EF-related processes, including attentional control and inhibitory regulation, help children manage competing information and sustain focus in communicative contexts. Together, these functions provide complementary scaffolds for language acquisition.

The persistence of these associations through 36 months underscores the continuity of cognitive–language coupling beyond infancy. Although environmental influences such as parent–child conversation and preschool experiences likely gain importance over time, the enduring predictive role of early memory and EF suggests that these capacities form part of the cognitive architecture supporting subsequent linguistic growth. These results thus point to a dynamic interplay between early cognitive foundations and environmental input throughout early development.

### 5.3. Temperament Findings

Analyses revealed that temperament dimensions showed consistent but modest associations with children’s language skills across the study period. At 12 months, higher surgency and effortful control significantly predicted both language comprehension and expression at 24 months, even after controlling for parental education, involvement, and responsiveness. These findings align with prior research showing that sociability and positive affectivity facilitate conversational engagement and vocabulary growth [7,8], whereas self-regulation supports sustained attention and the effective use of linguistic input [6,34]. In contrast, negative affectivity was not significantly related to language outcomes, which may reflect the buffering role of culturally responsive caregiving practices in Taiwanese families or the instability of affective traits at this age.

Temperament measured at 24 months continued to show small but significant associations with language skills at 36 months. Both surgency and effortful control remained positive predictors for comprehension and expression, though effect sizes were smaller (*β* ≈ 0.04–0.07). This pattern suggests that temperamental influences persist beyond infancy but gradually decline as language development becomes increasingly shaped by cumulative environmental input, such as parental responsiveness and the transition to preschool. Contextual factors specific to the Taiwanese setting may also contribute; around age three, many children enter structured childcare environments where enriched language experiences may attenuate individual differences linked to temperament.

Taken together, these results suggest that specific temperament dimensions—particularly surgency and effortful control—facilitate early language acquisition during the transition from infancy to toddlerhood by supporting children’s engagement in language-rich interactions. However, their unique effects appear to diminish as core linguistic skills consolidate and external environments become more uniformly stimulating. Future research should examine whether these traits predict higher-level language or literacy outcomes in later childhood and how cultural contexts moderate these developmental pathways.

### 5.4. Contribution

Despite these developmental variations, the findings provide community-based evidence from Taiwan that early memory and EF serve as observable indicators of early cognitive development, with predictive value for language outcomes during a critical window of growth. By linking insights from small-scale experimental studies with large-scale longitudinal data, this study demonstrates that everyday cognitive behaviors can meaningfully capture developmental processes underlying early learning. Importantly, the use of parent-reported observations offered a low-cost, widely applicable, and ecologically valid approach to assessing children’s real-world cognitive functioning.

For early childhood education and intervention, these findings emphasize the importance of attending to behaviors that reflect memory and EF—such as recalling familiar routines, sustaining attention during play, or inhibiting prohibited actions—as early indicators of children’s language trajectories. Because these behaviors can be observed in naturalistic classroom and home settings, they represent accessible cues for teachers and caregivers without the need for specialized testing. Identifying children who show early difficulties in these domains may help practitioners anticipate later language delays and provide targeted support at an earlier stage.

Observable behaviors related to memory and EF can also inform the design of play-based learning activities that foster both cognitive and language skills. In addition, such indicators provide a clear framework for communicating with parents about the cognitive foundations of language development.

Finally, incorporating cognitive indicators into developmental screening protocols could enhance existing practices that often focus solely on language milestones. Assessing both EF and memory in infancy and toddlerhood may enable educators and clinicians to design more holistic intervention strategies that strengthen foundational cognitive processes alongside language input. This integrated approach has the potential to improve outcomes for children at risk of language delays, particularly in settings where resources for specialized assessment are limited. Embedding such cognitive indicators into early education and intervention systems may further promote alignment between educational and clinical practices, ensuring that children’s developmental needs are addressed in a comprehensive and coordinated manner. At the policy level, incorporating cognitive indicators into national early childhood screening and curriculum frameworks could strengthen early identification systems and support evidence-based program design.

### 5.5. Limitations

#### 5.5.1. General Limitation

Several limitations should be acknowledged. First, measures of memory, EF, and language were derived from brief caregiver reports. Although reliability was acceptable, more fine-grained and performance-based assessments could capture individual differences with greater precision. Second, the study did not include direct physiological or neurodevelopmental indicators, which limits the ability to substantiate the proposed roles of hippocampal- and prefrontal-related processes. Third, although the analytic models accounted for key covariates, unmeasured environmental factors—such as preschool quality or peer interactions—may also shape language trajectories and merit further investigation.

#### 5.5.2. Limitations Related to Attrition

Although attrition across KIT waves was modest (<4%), analyses indicated that families who discontinued participation differed systematically from those who remained in the study, particularly on parental factors such as responsiveness, father involvement, and parental education (see Appendix B; full results in Supplementary Attrition Analysis). In contrast, no significant differences were observed on child-level variables, including temperament, cognition, and language. This pattern suggests that selective attrition may have introduced bias with respect to family-level influences, potentially leading to over- or underestimation of parental effects on children’s development. While the large sample and consistent results across models mitigate these concerns, caution is warranted when generalizing to populations with different distributions of parental resources. In particular, selective attrition may limit the generalizability of findings to families with higher socioeconomic and parenting resources. Nonetheless, the national scope of the KIT cohort and the robustness of the statistical results provide confidence in the validity of the observed patterns.

#### 5.5.3. Cultural Specificity and Cross-Cultural Considerations

These findings were derived from Taiwanese children within a specific sociocultural context. Cultural values, caregiving norms, and language exposure patterns may influence how early cognitive skills—such as memory and EF—relate to subsequent language development. Therefore, the generalizability of these associations to other populations and linguistic environments remains to be tested. Future cross-cultural and cross-linguistic research could clarify whether the developmental pathways linking cognition and language operate similarly across diverse cultural settings.

### 5.6. Future Directions

Future research could extend this work in several directions.

First, combining large-scale longitudinal datasets with smaller, targeted subsamples that include physiological or developmental measures would allow researchers to link population-level behavioral patterns with processes identified in controlled laboratory studies. Such integrative approaches may clarify how early cognitive functions associated with memory and executive control operate across diverse populations.

Second, refining behavioral assessments with age-appropriate, performance-based tasks would enhance sensitivity to developmental change and provide richer insight into within-child variability over time.

Third, future studies could examine potential mediating and moderating factors—such as caregiver responsiveness, bilingual exposure, and child temperament—to identify pathways through which early cognitive abilities shape later language outcomes.

Fourth, cross-cultural and cross-linguistic comparisons are needed to test whether the observed relations among early memory, executive function, and language generalize across different sociocultural contexts. These studies could explore how variations in parenting goals, interaction styles, and early educational experiences influence the developmental interplay between cognition and language. Including culturally sensitive measures of caregiver behavior and child engagement would further clarify whether the developmental mechanisms identified in Taiwanese toddlers are universal or context-specific.

Finally, extending longitudinal follow-up into the preschool and school years would reveal whether early cognitive abilities exert long-term effects on language development or whether environmental influences increasingly dominate as children grow older.

## Figures and Tables

**Table 1:** Descriptive statistics for cognitive and language measures at 36 months in the Kids in Taiwan (KIT) cohort.

Variable	*N*	*M*	*SD*
36 m Memory	6409	2.66	0.53
36 m Executive Function	6409	2.87	0.64
36 m Language Comprehension	6408	3.28	0.47
36 m Language Expression	6408	3.39	0.64

Note. *N*s reflect valid responses for each measure at 36 months. Means (*M*) and standard deviations (*SD*) are reported for continuous variables (memory, executive function, language comprehension, and language expression). Small variations in *N* across tables reflect item-level missingness; all participants belong to the 36-month cohort.

**Table 2:** Demographic characteristics of participants at 36 months.

Variable	Label	Frequency	Percentage (%)
Gender	Male	3411	51.30
	Female	3241	48.70
Paternal education level	Senior high school or below	1821	28.40
	Junior college	682	10.60
	College/University	2679	41.80
	Master’s degree or above	1228	19.20
Maternal education level	Senior high school or below	1595	24.70
	Junior college	711	11.00
	College/University	3360	51.90
	Master’s degree or above	809	12.50

Note. *N* = 6652. Small variations in *N* across Table 1 and Table 2 reflect item-level missingness; all participants belong to the 36-month cohort. Values are percentages unless otherwise noted.

**Table 3:** Means, Standard Deviations, Ranges, and Correlations of Study Variables.

Construct	Range	*M*	*SD*	ME12	EF12	LC12	LE12	ME24	EF24	LC24	LE24	ME36	EF36	LC36
ME12	1–4	2.44	0.31											
EF12	1–4	2.40	0.42	0.531 ***										
LC12	1–4	2.53	0.76	0.547 ***	0.432 ***									
LE12	1–4	1.05	0.16	0.210 ***	0.233 ***	0.241 **								
ME24	1–4	3.21	0.35	0.405 ***	0.305 ***	0.322 ***	0.154 ***							
EF24	1–4	3.36	0.44	0.337 ***	0.343 ***	0.273 ***	0.103 ***	0.512 ***						
LC24	1–4	3.72	0.45	0.357 ***	0.260 ***	0.331 ***	0.105 ***	0.507 ***	0.445 ***					
LE24	1–4	2.70	0.86	0.343 ***	0.263 ***	0.315 ***	0.154 ***	0.690 ***	0.410 ***	0.517 ***				
ME36	1–4	3.28	0.47	0.025 *	0.001	−0.002	−0.003	−0.008	−0.004	−0.037 *	−0.028 *			
EF36	1–4	3.40	0.64	0.256 ***	0.249 ***	0.200 ***	0.112 ***	0.339 ***	0.447 ***	0.266 ***	0.288 ***	0.012		
LC36	1–4	2.66	0.53	−0.008	0.021	0.018	−0.005	−0.014	−0.010	0.011	−0.013	0.054 ***	0.000	
LE36	1–4	2.87	0.64	−0.002	0.025 *	0.023	0.012	−0.016	−0.003	0.011	−0.009	−0.001	0.006	0.757 **

Note. *N* = 6652. ME = memory; EF = executive function; LC = language comprehension; LE = language expression. * *p* < 0.05. ** *p* < 0.01, *** *p* < 0.001.

**Table 4:** Hierarchical regression results for predicting language comprehension at 24 months.

Variable	B	95% CI for B		SE B	β	*R* ^2^	Δ*R*^2^
		LL	UL				
Step 1						0.053	0.053 ***
Constant	−2.153	−2.433	−1.873	0.143			
Paternal education (12 m)	0.032	0.005	0.059	0.014	0.037 *		
Maternal education (12 m)	0.054	0.025	0.083	0.015	0.058 ***		
Paternal involvement (12 m)	0.075	0.042	0.108	0.017	0.062 ***		
Maternal involvement (12 m)	0.070	0.005	0.135	0.033	0.030 *		
Parental responsiveness (12 m)	0.346	0.287	0.405	0.030	0.164 ***		
Step 2						0.154	0.101 ***
Constant	−2.046	−2.342	−1.750	0.151			
Paternal education (12 m)	0.029	0.004	0.054	0.013	0.035 *		
Maternal education (12 m)	0.063	0.036	0.090	0.014	0.068 ***		
Paternal involvement (12 m)	0.048	0.017	0.079	0.016	0.040 **		
Maternal involvement (12 m)	0.039	−0.022	0.100	0.031	0.017		
Parental responsiveness (12 m)	0.180	0.123	0.237	0.029	0.086 ***		
Child gender	0.067	0.018	0.116	0.025	0.033 **		
Surgency (12 m)	0.073	0.042	0.104	0.016	0.066 ***		
Effortful control (12 m)	0.101	0.062	0.140	0.020	0.075 ***		
Negative affectivity (12 m)	0.018	−0.011	0.047	0.015	0.015		
Language comprehension (12 m)	0.253	0.226	0.280	0.014	0.254 ***		
Step 3						0.177	0.024 ***
Constant	−3.136	−3.475	−2.797	0.173			
Paternal education (12 m)	0.028	0.003	0.053	0.013	0.033 *		
Maternal education (12 m)	0.055	0.028	0.082	0.014	0.059 ***		
Paternal involvement (12 m)	0.039	0.008	0.070	0.016	0.032 *		
Maternal involvement (12 m)	0.028	−0.033	0.089	0.031	0.012		
Parental responsiveness (12 m)	0.136	0.079	0.193	0.029	0.065 ***		
Child gender	0.054	0.007	0.101	0.024	0.027 *		
Surgency (12 m)	0.039	0.008	0.070	0.016	0.035 *		
Effortful control (12 m)	0.055	0.014	0.096	0.021	0.041 *		
Negative affectivity (12 m)	0.013	−0.016	0.042	0.015	0.011		
Language comprehension (12 m)	0.171	0.142	0.200	0.015	0.172 ***		
Memory (12 m)	0.614	0.510	0.718	0.053	0.187 ***		
Executive function (12 m)	0.058	−0.016	0.132	0.038	0.024		

Note. CI = confidence interval; LL = lower limit; UL = upper limit. * *p* < 0.05. ** *p* < 0.01. *** *p* < 0.001.

**Table 5:** Hierarchical regression analyses predicting expressive language at 24 months.

Variable	B	95% CI for B		SE B	β	*R* ^2^	Δ*R*^2^
		LL	UL				
Step 1						0.082	0.082 ***
Constant	−2.479	−2.755	−2.203	0.141			
Paternal education (12 m)	0.085	0.060	0.110	0.013	0.100 ***		
Maternal education (12 m)	0.088	0.059	0.117	0.015	0.095 ***		
Paternal involvement (12 m)	0.124	0.091	0.157	0.017	0.101 ***		
Maternal involvement (12 m)	0.052	−0.011	0.115	0.032	0.022		
Parental responsiveness (12 m)	0.303	0.244	0.362	0.030	0.144 ***		
Step 2						0.164	0.082 ***
Constant	−3.244	−3.530	−2.958	0.146			
Paternal education (12 m)	0.081	0.056	0.106	0.013	0.096 ***		
Maternal education (12 m)	0.083	0.056	0.110	0.014	0.089 ***		
Paternal involvement (12 m)	0.097	0.066	0.128	0.016	0.079 ***		
Maternal involvement (12 m)	0.035	−0.026	0.096	0.031	0.015		
Parental responsiveness (12 m)	0.189	0.132	0.246	0.029	0.090 ***		
Child gender	0.329	0.280	0.378	0.025	0.165 ***		
Surgency (12 m)	0.117	0.086	0.148	0.016	0.107 ***		
Effortful control (12 m)	0.168	−0.204	0.540	0.019	0.124 ***		
Negative affectivity (12 m)	0.014	−0.015	0.043	0.015	0.011		
Language expression (12 m)	0.097	0.072	0.122	0.013	0.097 ***		
Step 3						0.201	0.037 ***
Constant	−4.277	−4.585	−3.969	0.157			
Paternal education (12 m)	0.080	0.056	0.104	0.012	0.094 ***		
Maternal education (12 m)	0.079	0.055	0.103	0.012	0.085 ***		
Paternal involvement (12 m)	0.087	0.056	0.118	0.016	0.071 ***		
Maternal involvement (12 m)	0.018	−0.041	0.077	0.030	0.008		
Parental responsiveness (12 m)	0.118	0.061	0.175	0.029	0.056 ***		
Child gender	0.303	0.256	0.350	0.024	0.152 ***		
Surgency (12 m)	0.065	0.034	0.096	0.016	0.059 ***		
Effortful control (12 m)	0.074	0.033	0.115	0.021	0.055 ***		
Negative affectivity (12 m)	0.005	−0.024	0.034	0.015	0.004		
Language expression (12 m)	0.067	0.042	0.092	0.013	0.066 ***		
Memory (12 m)	0.669	0.573	0.765	0.049	0.203 ***		
Executive function (12 m)	0.114	0.040	0.188	0.038	0.048 **		

Note. CI = confidence interval; LL = lower limit; UL = upper limit. ** *p* < 0.01. *** *p* < 0.001.

**Table 6:** Hierarchical regression analyses predicting language comprehension at 36 months.

Variable	B	95% CI for B		SE B	β	*R* ^2^	Δ*R*^2^
		LL	UL				
Step 1						0.026	0.026 ***
Constant	−1.342	−1.605	−1.079	0.134			
Paternal education (24 m)	0.004	−0.021	0.029	0.013	0.005		
Maternal education (24 m)	−0.005	−0.032	0.022	0.014	−0.005		
Paternal involvement (24 m)	0.102	0.071	0.133	0.016	0.084 ***		
Maternal involvement (24 m)	0.051	−0.010	0.112	0.031	0.022		
Parental responsiveness (24 m)	0.223	0.168	0.278	0.028	0.107 ***		
Step 2						0.152	0.126 ***
Constant	−1.509	−1.803	−1.215	0.150			
Paternal education (24 m)	−0.003	−0.027	0.021	0.012	−0.004		
Maternal education (24 m)	−0.025	−0.050	0.000	0.013	−0.028		
Paternal involvement (24 m)	0.056	0.027	0.085	0.015	0.046 ***		
Maternal involvement (24 m)	0.028	−0.029	0.085	0.029	0.012		
Parental responsiveness (24 m)	0.016	−0.039	0.071	0.028	0.008		
Child gender	0.080	0.035	0.125	0.023	0.040 **		
Surgency (24 m)	0.138	0.101	0.175	0.019	0.098 ***		
Effortful control (24 m)	0.186	0.147	0.225	0.020	0.131 ***		
Negative affectivity (24 m)	−0.012	−0.039	0.015	0.014	−0.010		
Language comprehension (24 m)	0.247	0.222	0.272	0.013	0.247 ***		
Step 3						0.181	0.029 ***
Constant	−2.898	−3.239	−2.557	0.174			
Paternal education (24 m)	−0.022	−0.046	0.002	0.012	−0.026		
Maternal education (24 m)	−0.041	−0.066	−0.016	0.013	−0.044 **		
Paternal involvement (24 m)	0.048	0.019	0.077	0.015	0.039 **		
Maternal involvement (24 m)	0.013	−0.042	0.068	0.028	0.006		
Parental responsiveness (24 m)	−0.015	−0.068	0.038	0.027	−0.007		
Child gender	0.029	−0.016	0.074	0.023	0.015		
Surgency (24 m)	0.102	0.065	0.139	0.019	0.072 ***		
Effortful control (24 m)	0.068	0.025	0.111	0.022	0.048 **		
Negative affectivity (24 m)	−0.003	−0.030	0.024	0.014	−0.003		
Language comprehension (24 m)	0.162	0.135	0.189	0.014	0.162 ***		
Memory (24 m)	0.449	0.365	0.533	0.043	0.159 ***		
Executive function (24 m)	0.270	0.199	0.341	0.036	0.119 ***		

Note. CI = confidence interval; LL = lower limit; UL = upper limit. ** *p* < 0.01. *** *p* < 0.001.

**Table 7:** Hierarchical regression analyses predicting expressive language at 36 months.

Variable	B	95% CI for B		SE B	β	*R* ^2^	Δ*R*^2^
		LL	UL				
Step 1						0.096	0.096 ***
Constant	−2.740	−2.991	−2.489	0.128			
Paternal education (24 m)	0.065	0.041	0.089	0.012	0.077 ***		
Maternal education (24 m)	0.094	0.069	0.119	0.013	0.103 ***		
Paternal involvement (24 m)	0.098	0.069	0.127	0.015	0.081 ***		
Maternal involvement (24 m)	0.063	0.004	0.122	0.030	0.027 *		
Parental responsiveness (24 m)	0.400	0.347	0.453	0.027	0.194 ***		
Step 2						0.320	0.223 ***
Constant	−2.139	−2.408	−1.870	0.137			
Paternal education (24 m)	0.029	0.007	0.051	0.011	0.035 *		
Maternal education (24 m)	0.053	0.029	0.077	0.012	0.059 ***		
Paternal involvement (24 m)	0.036	0.011	0.061	0.013	0.030 *		
Maternal involvement (24 m)	0.039	−0.012	0.090	0.026	0.017		
Parental responsiveness (24 m)	0.134	0.085	0.183	0.025	0.065 ***		
Child gender	0.059	0.018	0.100	0.021	0.030 *		
Surgency (24 m)	0.085	0.052	0.118	0.017	0.061 ***		
Effortful control (24 m)	0.148	0.115	0.181	0.017	0.105 ***		
Negative affectivity (24 m)	0.014	−0.010	0.038	0.012	0.012		
Language expression (24 m)	0.420	0.396	0.444	0.012	0.424 ***		
Step 3						0.338	0.018 ***
Constant	−3.458	−3.793	−3.123	0.171			
Paternal education (24 m)	0.020	−0.002	0.042	0.011	0.023		
Maternal education (24 m)	0.044	0.020	0.068	0.012	0.048 ***		
Paternal involvement (24 m)	0.032	0.007	0.057	0.013	0.026 *		
Maternal involvement (24 m)	0.028	−0.021	0.077	0.025	0.012		
Parental responsiveness (24 m)	0.117	0.070	0.164	0.024	0.057 ***		
Child gender	0.039	−0.002	0.080	0.021	0.020		
Surgency (24 m)	0.057	0.024	0.090	0.017	0.041 **		
Effortful control (24 m)	0.060	0.023	0.097	0.019	0.042 **		
Negative affectivity (24 m)	0.017	−0.007	0.041	0.012	0.015		
Language expression (24 m)	0.327	0.300	0.354	0.014	0.330 ***		
Memory (24 m)	0.386	0.300	0.472	0.044	0.137 ***		
Executive function (24 m)	0.219	0.156	0.282	0.032	0.096 ***		

Note. CI = confidence interval; LL = lower limit; UL = upper limit. * *p* < 0.05. ** *p* < 0.01. *** *p* < 0.001.

## Data Availability

The data used in this study were obtained from the Kids in Taiwan (KIT) project, a national longitudinal database established by the KIT research team. The anonymized secondary data analyzed in this study were accessed through the Survey Research Data Archive (SRDA) of Academia Sinica (https://srda.sinica.edu.tw/) after registration and application. Information about the KIT project is available at https://kit.hdfs.ntnu.edu.tw/CN/index.aspx (accessed on 22 March 2024). No new data were created or analyzed in this study.

## Abbreviations

The following abbreviations are used in the present study:

KIT	Kids in Taiwan: National Longitudinal Study of Child Development & Care
EF	Executive Function
ME	memory
LC	language comprehension
LE	language expression
12 m	12 months of age
24 m	24 months of age
36 m	36 months of age

## Appendix A. Summary of Study Variables and Measures

**Table A1:** Summary of Study Variables and Reliability.

Construct	Example Item(s)	Reliability (Cronbach’s α)
Maternal/Paternal Involvement	“Provides for the child’s basic daily needs (e.g., food, clothing)”, “Teaches the child daily routines”, “ Helps the child with learning activities”, “Plays with the child”	α = 0.81
Parental Responsiveness	“Responds verbally when the child makes sounds or speaks”, “Kisses or hugs the child”, “Talks with the child while doing chores”	α = 0.52~0.58
Surgency	“Joins new activities immediately”, “Plays with different people at familiar gatherings”	α = 0.60~0.63
Effortful Control	“Waits patiently for desired objects when reminded by an adult”, “Adjusts behavior for safety or environmental demands when reminded (e.g., walks carefully in a place with fragile items)”	α = 0.63~0.85
Negative Affectivity	“Cries when not given attention”, “Gets angry or throws tantrums easily”	α = 0.60~0.68
Memory	12 & 24 m: “Remembers familiar storybooks”, “Recalls caregiver’s name” 36 m: “Recognizes previously read or heard storybooks”, “Recalls a home or family telephone number”	α = 0.95
Executive Function	12 & 24 m: “Stops a prohibited action when told”, “Stops playing with an object when persuaded to do something else” 36 m: “Slows down when reminded to do things better”, “Lowers voice in public when asked and maintains for several minutes”	α = 0.95
Language Comprehension	12 & 24 m: “Understands simple instructions”, “Recognizes familiar objects by name” 36 m: “Understands and follows two or more oral instructions in sequence”, “Understands simple jokes or word play”	α = 0.61~0.73
Language Expression	12 & 24 m: “Combines two words”, “Uses descriptive words in daily life” 36 m: “Answers ‘What’ questions”, “Answers ‘Why’ questions”	α = 0.75~0.82

Note. Language and cognitive abilities were rated on a 4-point Likert-type scale (Response Scale: 1 = Not at all able; 2 = Occasionally able; 3 = Fairly proficient; 4 = Highly proficient). To ensure developmental appropriateness, different items were used at 12–24 months and 36 months. Criterion-related validity was examined in relation to the Comprehensive Developmental Inventory for Infants and Toddlers (CDIIT) [35]). For language development: 12–24 months: Language comprehension (r = 0.73); Language expression (r = 0.82). 36 months: Language comprehension (r = 0.61); Language expression (r = 0.75). For cognitive development, criterion-related validity was examined in relation to the CDIIT Cognitive Concept scale [35]. The total cognitive score at 36 months was significantly correlated with the CDIIT Cognitive Concept score (r = 0.63), and the total cognitive score at 12–24 months showed a significant correlation of r = 0.41. Department of Special Education, National Taiwan Normal University. The Memory and Executive Function (EF) subscales comprised 17 and 6 items, respectively, at both 12 and 24 months. Internal consistency estimates (Cronbach’s α) indicated satisfactory reliability for this age range: at 12 months, α = 0.77 for Memory and α = 0.62 for EF; at 24 months, α = 0.78 for Memory and α = 0.69 for EF. The items listed in this appendix represent example items only; the full scales include a broader range of age-appropriate behaviors covering domains such as object permanence, deferred imitation, and inhibitory control. Temperament variables were assessed with parent ratings of surgency, effortful control, and negative affectivity, rated on a 5-point Likert-type scale based on parents’ observations of their child’s typical behaviors, ranging from 1 (Never—the child has never shown the behavior) to 5 (Always—the child consistently shows the behavior). 1 = Never—The child has never exhibited the behavior. 2 = Rarely—The child shows the behavior only once or twice on rare occasions. 3 = Sometimes—The child shows the behavior inconsistently; sometimes yes, sometimes no. = Often—The child shows the behavior frequently. 5 = Always—The child consistently shows the behavior. Cronbach’s α coefficients for temperament subscales were as follows: 12–24 months: Surgency/Extraversion (α = 0.60), Effortful Control (α = 0.85), Negative Affectivity (α = 0.68); 36 months: Surgency/Extraversion (α = 0.66), Effortful Control (α = 0.63), Negative Affectivity (α = 0.60). Parental background variables included education level, involvement in child-rearing, and parental responsiveness. (1) Parental education level was coded on a 6-point ordinal scale: 1 = Elementary school or below, 2 = Junior high school, 3 = Senior high school or vocational school, 4 = Junior college, 5 = University or technical college, 6 = Master’s degree or above. For analysis, the variable was treated as continuous, and descriptive statistics are reported as means and standard deviations. (2) Family-related caregiving variables included Parental Involvement and Parental Responsiveness, both assessed over the past three months using a 4-point Likert-type frequency scale. Parental Involvement was rated separately for fathers and mothers, based on their participation in children’s daily caregiving activities. Parental Responsiveness was rated as a combined measure of parents’ verbal and emotional responses to the child, with items such as “I respond verbally when my child makes sounds or speaks,” “I kiss or hug my child,” and “I talk with my child while doing chores.” Response options for both constructs were: 1 = Rarely (never or less than once per week on average), 2 = Sometimes (1–2 times per week), 3 = Often (3–4 times per week), 4 = Very often (5–7 times per week). For Parental Involvement and Responsiveness, if the child did not have a father or mother, respondents selected “Not applicable” for the related items.

## Appendix B. Supplementary Attrition Analysis Results

Comparison of retained and attrited participants on family background, child temperament, and cognition/language variables. Values are presented as means (standard deviations) for continuous variables and percentages for categorical variables.

**Table A2:** Comparison of Retained and Attrited Participants from 12 to 24 months.

Variable	Retained *M (SD)*/%	Attrited *M (SD)*/%	*t*(df)/*χ^2^*(df)	*p*-Value	Effect Size
Paternal education (years)	4.45 (1.18)	4.23 (1.33)	*t*(304) = 2.72	<0.001	*d* = 0.17
Maternal education (years)	4.47 (1.08)	4.18 (1.28)	*t*(309) = 3.73	<0.001	*d* = 0.24
Paternal involvement	3.09 (0.82)	2.95 (0.82)	*t*(6725) = 1.88	0.060	*d* = 0.17
Maternal involvement	3.79 (0.44)	3.75 (0.50)	*t*(364) = 1.51	0.133	*d* = 0.08
Parental responsiveness	3.70 (0.48)	3.59 (0.57)	*t*(373) = 3.47	0.001	*d* = 0.21
Child sex (male, %)	51.20%	51.10%	*χ*^2^(1) = 0.003	0.960	*φ* = 0.02
Surgency	3.27 (0.92)	3.10 (0.95)	*t*(6866) = 3.37	0.001	*d* = 0.18
Effortful control	2.73 (0.74)	2.67 (0.75)	*t*(6871) = 1.41	0.160	*d* = 0.08
Negative affectivity	3.58 (0.82	3.52 (0.82)	*t*(6872) = 1.32	0.186	*d* = −0.07
Memory	2.44 (0.31)	2.44 (0.34)	*t*(6870) = 0.13	0.897	*d* = 0.00
Executive function	2.40 (0.42)	2.45 (0.49)	*t*(374) = 2.30	0.021	*d* = −0.11
Language comprehension	2.53 (0.77)	2.62 (0.81)	*t*(379) = 1.96	0.050	*d* = −0.11
Language expression	1.05 (0.16)	1.07 (0.17)	*t*(379) = 1.77	0.077	*d* = −0.12

Note. Values are presented as means (standard deviations) for continuous variables and as percentages for categorical variables (e.g., child sex). Independent-samples t-tests were used for continuous variables; when Levene’s test indicated unequal variances, Welch’s adjusted degrees of freedom are reported. Effect sizes for continuous variables are expressed as Cohen’s d (0.2 = small, 0.5 = medium, 0.8 = large). For categorical variables, chi-square tests were used, with Cramer’s V reported as the effect size (0.1 = small, 0.3 = medium, 0.5 = large). Positive d values indicate higher means in the retained group. The effect sizes of the differences between the retained and attrited groups were generally small. Across all variables, Cohen’s *d* values ranged from –0.12 to 0.24, with the majority falling below 0.20. According to conventional benchmarks, values of *d* ≈ 0.20 represent small effects, *d* ≈ 0.50 represent medium effects, and *d* ≈ 0.80 represent large effects. In the present analyses, most effects were negligible to small, indicating that attrition was unlikely to bias the results in a meaningful way. For the categorical variable, the effect size was *φ* = 0.02, which also falls within the small range [36].

**Table A3:** Comparison of Retained and Attrited Participants from 24 to 36 Months.

Variable	Retained *M (SD)*	Attrited M (SD)	*t*(df)/*χ^2^*(df)	*p*	Effect Size
Paternal education (years)	4.46 (1.17)	4.28 (1.30)	*t*(296) = 3.41	0.001	*d* = 0.15
Maternal education (years)	4.48 (1.10)	4.31 (1.26)	*t*(303) = 3.68	<0 0.001	*d* = 0.15
Paternal involvement	3.12 (0.81)	3.07 (0.84)	*t*(292) = 3.42	0.001	*d* = 0.06
Maternal involvement	3.81 (0.43)	3.80 (0.49)	*t*(300) = 1.49	0.137	*d* = 0.04
Parental responsiveness	3.71 (0.48)	3.70 (0.49)	*t*(305) = 3.47	0.001	*d* = 0.08
Child sex (male %)	51.10%	51.20%	*χ^2^*(1) = 0.003	0.960	*φ* = 0.02
Surgency	3.88 (0.71)	3.85 (0.69)	*t*(6769) = 0.69	0.487	*d* = 0.04
Effortful control	3.67 (0.70)	3.65 (0.70)	*t*(6770) = 0.55	0.582	*d* = 0.03
Negative affectivity	3.51 (0.86)	3.60 (0.75)	*t*(316) = −1.97	0.050	*d* = −0.11
Cognitive memory	3.21 (0.35)	3.18 (0.39)	*t*(304) = 1.30	0.194	*d* = 0.09
Cognitive EF	3.38 (0.44)	3.38 (0.47)	*t*(305) = −0.74	0.459	*d* = 0.05
Language comprehension	3.72 (0.45)	3.72 (0.46)	t(6771) = −0.20	0.839	*d* = −0.01
Language expression	2.70 (0.86)	2.72 (0.95)	*t*(303) = −0.42	0.672	*d* = −0.03

Note. Values are presented as means (standard deviations) for continuous variables and as percentages for categorical variables (e.g., child sex). Independent-samples t-tests were used for continuous variables; when Levene’s test indicated unequal variances, Welch’s adjusted degrees of freedom are reported. Effect sizes for continuous variables are expressed as Cohen’s *d* (0.2 = small, 0.5 = medium, 0.8 = large). For categorical variables, chi-square tests were used, with Cramer’s *V* reported as the effect size (0.1 = small, 0.3 = medium, 0.5 = large). Positive d values indicate higher means in the retained group. The effect sizes of the differences between the retained and attrited groups were generally small. Across all variables, Cohen’s *d* values ranged from –0.11 to 0.15, with the majority falling below 0.20. According to conventional benchmarks, values of *d* ≈ 0.20 represent small effects, *d* ≈ 0.50 represent medium effects, and *d* ≈ 0.80 represent large effects. In the present analyses, most effects were negligible to small, indicating that attrition was unlikely to bias the results in a meaningful way. For the categorical variable, the effect size was *φ* = 0.02, which also falls within the small range [36].
